# Highly Pathogenic Avian Influenza H5N8 Clade 2.3.4.4b in Germany in 2016/2017

**DOI:** 10.3389/fvets.2017.00240

**Published:** 2018-01-24

**Authors:** Anja Globig, Christoph Staubach, Carola Sauter-Louis, Klaas Dietze, Timo Homeier-Bachmann, Carolina Probst, Jörn Gethmann, Klaus R. Depner, Christian Grund, Timm C. Harder, Elke Starick, Anne Pohlmann, Dirk Höper, Martin Beer, Thomas C. Mettenleiter, Franz J. Conraths

**Affiliations:** ^1^Friedrich-Loeffler-Institut, Federal Research Institute for Animal Health, Greifswald-Insel Riems, Germany

**Keywords:** highly pathogenic avian influenza, H5N8, clade 2.3.4.4b, Germany, wild water birds, outbreak investigations, primary incursion, farm-to-farm spread

## Abstract

Here, we report on the occurrence of highly pathogenic avian influenza (HPAI) H5Nx clade 2.3.4.4b in Germany. Between November 8, 2016, and September 30, 2017, more than 1,150 cases of HPAI H5Nx clade 2.3.4.4b in wild birds and 107 outbreaks in birds kept in captivity (92 poultry holdings and 15 zoos/animal parks) were reported in Germany. This HPAI epidemic is the most severe recorded in Germany so far. The viruses were apparently introduced by migratory birds, sparking an epidemic among wild birds across Germany with occasional incursions into poultry holdings, zoos and animal parks, which were usually rapidly detected and controlled by stamping out. HPAI viruses (mainly subtype H5N8, in a few cases also H5N5) were found in dead wild birds of at least 53 species. The affected wild birds were water birds (including gulls, storks, herons, and cormorants) and scavenging birds (birds of prey, owls, and crows). In a number of cases, substantial gaps in farm biosecurity may have eased virus entry into the holdings. In a second wave of the epidemic starting from February 2017, there was epidemiological and molecular evidence for virus transmission of the infections between commercial turkey holdings in an area of high poultry density, which caused approximately 25% of the total number of outbreaks in poultry. Biosecurity measures in poultry holdings should be adapted. This includes, *inter alia*, wearing of stable-specific protective clothing and footwear, cleaning, and disinfection of equipment that has been in contact with birds and prevention of contacts between poultry and wild water birds.

## Introduction

Avian Influenza is an infectious disease of poultry caused by influenza A viruses, which are enveloped viruses of the family *Orthomyxoviridae* with a segmented single-stranded RNA genome. These viruses occur in two pathogenicity variants (low/highly pathogenic) and a multitude of different subtypes. Wild water birds (*Anseriformes*) as well as gulls, terns, and wader birds (*Charadriiformes*) are regarded as the natural reservoir for all low pathogenic avian influenza viruses (LPAIVs), i.e., viruses of the subtypes H1–H16 and N1–N9. While LPAIV of the subtypes H5 and H7 may cause almost no or only mild disease in domestic poultry, these subtypes have the capacity to evolve spontaneously into highly pathogenic forms [highly pathogenic avian influenza viruses (HPAIVs)]. The underlying mutational steps seem to be associated with adaptation to domestic poultry after transmission of the low pathogenic progenitors from wild birds (1). The highly pathogenic form clinically manifests itself in poultry as fowl plague, which causes drastic losses especially in turkeys and chickens. In ducks and geese, however, the clinical signs of an HPAIV infection may be mild, and mortality can be considerably lower than in turkeys and chickens. Therefore, HPAIV may circulate in waterfowl undetected, whereas mortality is always very high in *Galliformes* [75–100% (2)].

Upon exposure to a high infectious dose, usually by direct contact to infected birds, some avian influenza viruses (AIVs) (e.g., HPAIV H5N1 and H5N6, LPAIV H7N9 in China, of which a HPAI variant has recently been detected) can be transmitted to humans and may cause fatal disease. Due to the segmented genome of influenza A viruses, new viruses can evolve, when simultaneous infections of a single host with different influenza A viruses allow mixing (reassortment) of the genome segments. Therefore, there is a permanent risk for the generation of novel influenza viruses with pandemic potential if different zoonotic influenza A virus strains cocirculate (3).

In 1996, a HPAIV of subtype H5N1 originating from geese (goose/Guangdong/96, gs/GD) in southern China caused outbreaks in chickens and disease in 18 humans with six fatalities. This virus evolved steadily during the following two decades into various phylogenetic clades, subtypes, and genotypes within the so-called gs/GD lineage. A combination of blanket vaccination of poultry against HPAI H5, trading at live bird markets and the traditional way of keeping waterfowl, for example, in rice fields, in contact to wild or feral water birds is a perfect source for the genesis, emergence, and evolution of new HPAIVs in large parts of Asia, especially in South East Asia. Migratory water birds mixing with poultry may contribute to the development of new viruses by reassortment and eventually give rise to intra- and intercontinental spread. Many of the gs/GD H5-descendants caused serious outbreaks of fowl plague in poultry in South East Asia and some were detected in Europe as well: in 2005/2006 (H5N1 clade 2.2), in 2010 (H5N1 clade 2.3.2.1c), and in 2014 (H5N8 clade 2.3.4.4a). This led to a massive increase of HPAI outbreaks worldwide since 1996 (4–6). Some, but not all of these HPAI H5 strains can also cause severe infections in humans. The generation of a potentially pandemic virus from this lineage that is able to spread within the human population is of worldwide concern and under careful observation. Genetic analysis and animal experiments showed that there was no indication of a zoonotic potential of the clade 2.3.4.4 H5N8a and b viruses (7) and no human infections with this virus have been reported so far. However, 2.3.4.4c H5N6 viruses, which have hitherto only been detected in South East Asia, bear a zoonotic potential (8).

In September 2016, the FAO released a risk alert about the potential westward spread of a novel HPAIV H5N8 of clade 2.3.4.4b, which was detected through surveillance of wild migratory birds in the Tyva Republic, Russian Federation, in June 2016 (9). Only one month later, Hungary and then Poland notified the first cases of HPAIV H5N8 clade 2.3.4.4b detection in dead wild birds (a swan in Hungary and ducks as well as gulls in Poland).

Here, we provide a brief account of the course of the HPAI epidemic that took place in wild and kept birds in Germany in 2016–2017.

## Materials and Methods

### Case and Outbreak Data

Records of cases of HPAIV infections in wild birds and HPAI outbreaks in kept birds in Germany, i.e., commercial and backyard poultry holdings as well as zoos, were obtained from the German National Animal Disease Data Base (10). In brief, all cases of HPAIV detection in wild and captive birds were submitted to the database by the competent veterinary authorities at the district level.

Records on HPAI cases in wild birds in Germany were retrieved from the “Wildvogelmonitoring-Datenbank”, the National Avian Influenza Data Base run by the Friedrich-Loeffler-Institut (11). Data on the type of surveillance (active or passive), the sampled wild bird species and the laboratory result were entered by the veterinary investigation centers of the respective federal states.

Data on outbreaks in poultry and cases in wild birds in Europe were obtained from the European Animal Disease Notification System^1^ and EMPRES Global Animal Disease Information System (FAO^2^). Data were analyzed in Excel spreadsheets (Microsoft Excel, 2016). Maps were created using ArcGIS software (ESRI, Redlands, CA, USA).

### Epidemiological Outbreak Investigations

Epidemiological outbreak investigations were conducted in affected poultry holdings and zoos according to Council Directive 2005/94/EC as previously described (12). In brief, data were obtained by on-site visits to the holdings and by structured interviews with farm or zoo managers, employees, and veterinarians who had visited the farm or zoo. Additional data were extracted from invoices, trade documents (purchase of poultry and feed), and stable records of the affected holdings if available. Touring records of the veterinarians and of vehicles (feed transports, rendering lorries, etc.) were checked for their potential role in virus introduction into the affected holdings.

## Results

### HPAI H5N8 Clade 2.3.4.4b Outbreaks in Europe and Germany

**Figure 1:**
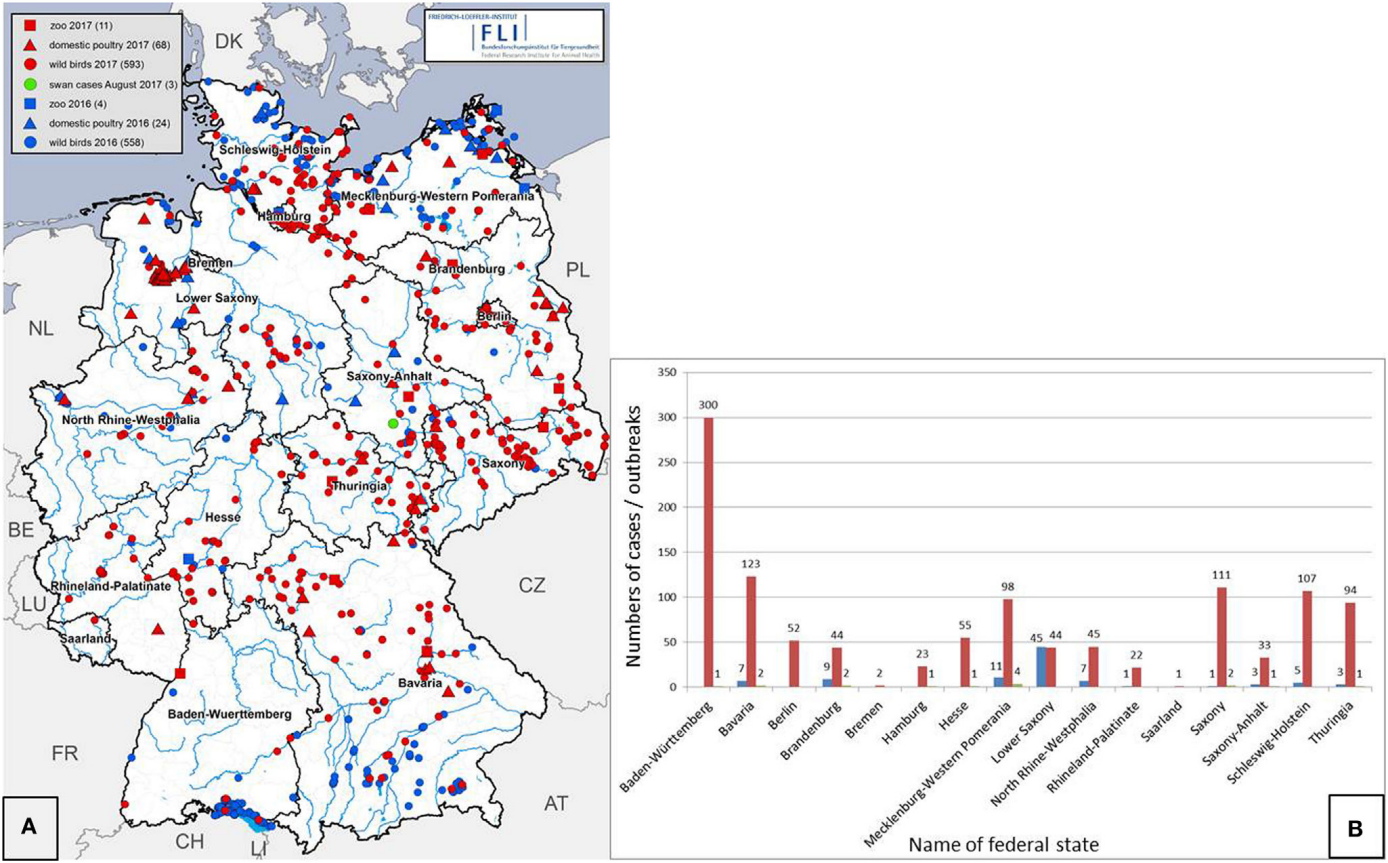
Reported highly pathogenic avian influenza (HPAI) clade 2.3.4.4b H5Nx cases in wild birds (points) and outbreaks in poultry holdings (triangles) and zoos (squares) in the German federal states in 2016 (blue) and 2017 (red). Green points refer to mute swans found HPAIV H5N8 infected in August 2017 **(A)**. Number of cases in wild birds (red) and outbreaks in poultry holdings (blue) and zoos (green) in each German federal state **(B)**.

On November 7, 2016, shortly after the first detection of HPAIV H5N8 clade 2.3.4.4b in Hungary and Poland, an increased mortality of uncertain cause was first reported in tufted ducks (*Aythya fuligula*) at Lake Constance in Baden-Württemberg, in the southwest of Germany. One day later, on November 8, 2016, HPAIV H5N8 was identified in wild birds (mostly tufted ducks) at Lake Constance as well as in tufted ducks found dead at Lake Plön in Schleswig-Holstein, northern Germany. Simultaneously, an increased number of wild water birds and sea gulls were found dead at the eastern coast of Schleswig-Holstein, around Lake Constance in Switzerland, Austria, and Germany (Bavaria and Baden-Württemberg) as well as at the Baltic Sea Coast in Mecklenburg-Western Pomerania, northeastern Germany (Figure 1A, blue points).

**Figure 2:**
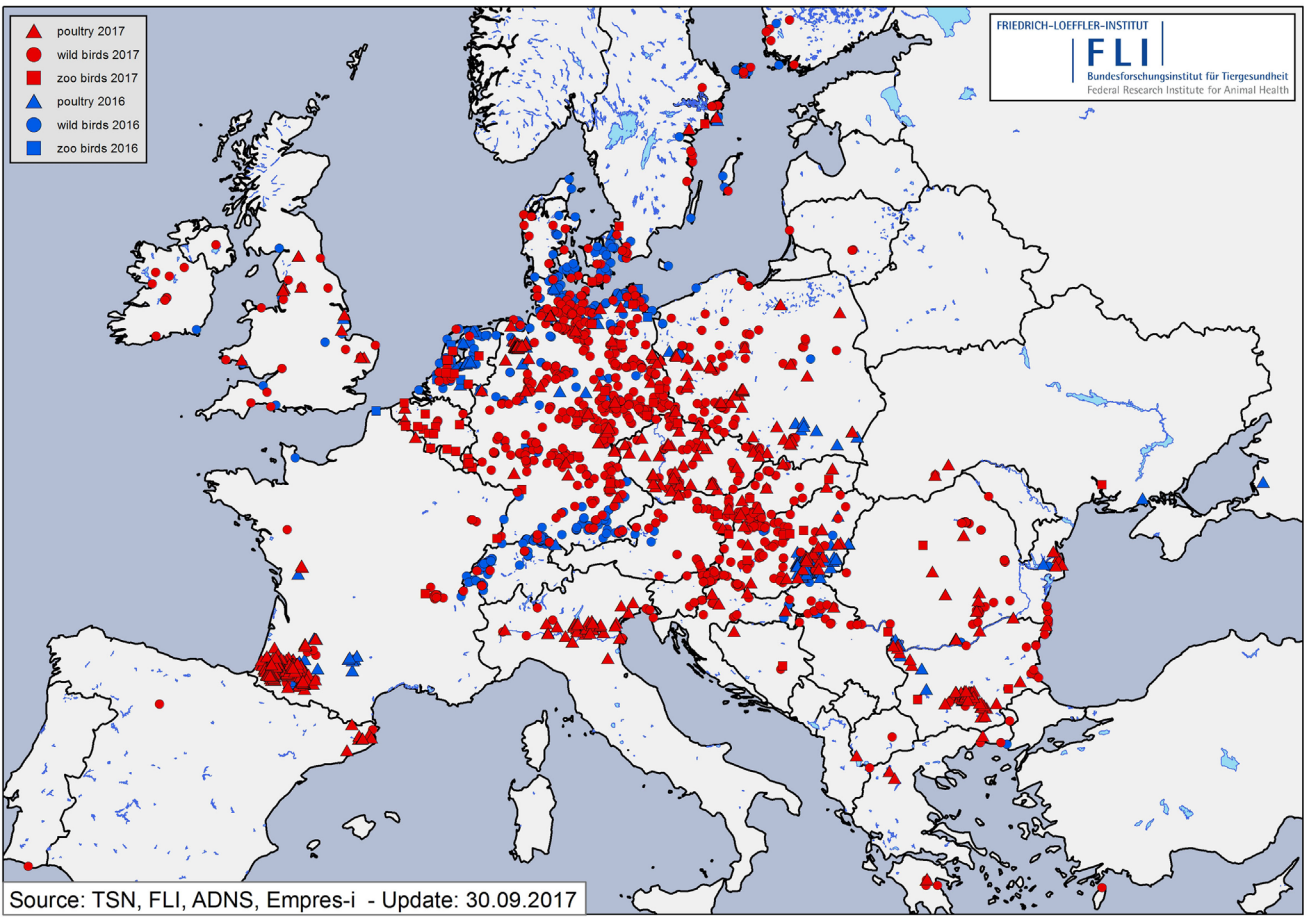
Distribution of reported highly pathogenic avian influenza clade 2.3.4.4b H5Nx cases in wild birds (points) and outbreaks in poultry holdings (triangles) and captive birds in zoos (squares) in 2016 (blue) and 2017 (red) in Europe.

Soon, the HPAI H5N8 infections widened to an epidemic across Germany (Figure 1A, red points) affecting mainly wild water birds of the orders *Anseriformes, Podicipediformes, Charadriiformes, Phalacrocoraciformes, Ardeiformes*, and *Ciconiiformes* overwintering at lakes and rivers or along the coast, and scavenging birds of the orders *Accipitriformes, Falconiformes*, and *Strigiformes* as well as in few cases also crows that had apparently fed on infected carcasses. The virus was isolated from at least 53 wild or feral bird species (Table 1). Almost all other European countries were affected by the epidemic as well (Figure 2).

**Table 1:** Species of wild or feral (marked with *) birds infected with HPAIV clade 2.3.4.4b H5N8/N5.

Species	Latin name
**Order *Anseriformes***	
**Diving ducks**	***Aythya***
Tufted duck	*Aythya fuligula*
Common pochard	*Aythya ferina*
Common goldeneye	*Bucephala clangula*
Red crested pochard	*Netta Rufina*
Greater scaup	*Aythya marila*
Common eider	*Somateria mollissima*
Common scoter	*Melanitta nigra*

**Dabbling ducks**	***Anas***
Mallard	*Anas platyrhynchos*
Northern pintail	*Anas acuta*
Gadwall	*Mareca strepera*
Eurasian wigeon	*Anas penelope*

**Perching ducks**	***Anatini***
Wood duck	*Aix sponsa*

**Ruddy ducks**	***Oxyura***
Ruddy duck*	*Oxyura jamaicensis*

**Shelducks**	***Tadorninae***
Common shelduck	*Tadorna tadorna*

	***Podiceps***
Great crested grebe	*Podiceps cristatus*
Red-necked grebe	*Podiceps grisegena*
Little grebe	*Tachybaptus ruficollis*

**Merganser**	***Mergus***
Merganser	*mergus*
Common merganser	*Mergus merganser*

**Goose**	
Greylag goose	*Anser anser*
Bean goose	*Anser fabalis*
Canada goose	*Branta canadensis*
White-fronted goose	*Anser albifrons*
Pink-footed goose	*Anser brachyrhynchus*
Barnacle goose	*Branta leucopsis*
Dark-bellied brant	*Branta bernicla*
Red-breasted goose*	*Branta ruficollis*
Lesser white-fronted goose	*Anser erythropus*

**Swans**	***Cygnus***
Mute swan	*Cygnus olor*
Black swan*	*Cygnus atratus*
Whooper swan	*Cygnus cygnus*

**Order *Charadriiformes***	
**Gulls**	***Laridae***
Black-headed gull	*Chroicocephalus ridibundus*
European herring gull	*Larus argentatus*
Great black-backed gull	*Larus marinus*
Mew gull	*Larus canus*
Little gull	*Hydrocoloeus minutus*
Lesser black-backed gull	*Larus fuscus*

**Sandpipers**	***Scolopacidae***
Red shank	*Tringa totanus*

**Order *Gruiformes***	
**Rail**	***Rallidae***
Common coot	*Fulica atra*

**Order *Ardeiformes***	
Grey heron	*Ardea cinerea*
Western great egret	*Ardea alba*

**Order *Accipitriformes***	
	***Accipitridae***
Common buzzard	*Buteo buteo*
Rough-legged buzzard	*Buteo lagopus*
White-tailed eagle	*Haliaeetus albicilla*
Northern goshawk	*Accipiter gentilis*
Eurasian sparrowhawk	*Accipiter nisus*

**Order *Phalacrocoraciformes***	
Great cormorant	*Phalacrocorax carbo*

**Order *Passeriformes***	
**Crows**	***Corvidae***
Carrion crow	*Corvus corone*
Magpie	*Pica pica*

**Order *Ciconiiformes***	
**Storks**	***Ciconiidae***
White stork	*Ciconia ciconia*

**Order *Falconiformes***	
**Falcons**	***Falco***
Peregrine falcon	*Falco peregrinus*

**Order *Strigiformes***	
**Owls**	***Strigidae***
Long-eared Owl	*Asio otus*
Tawny owl	*Strix aluco*

**Figure 3:**
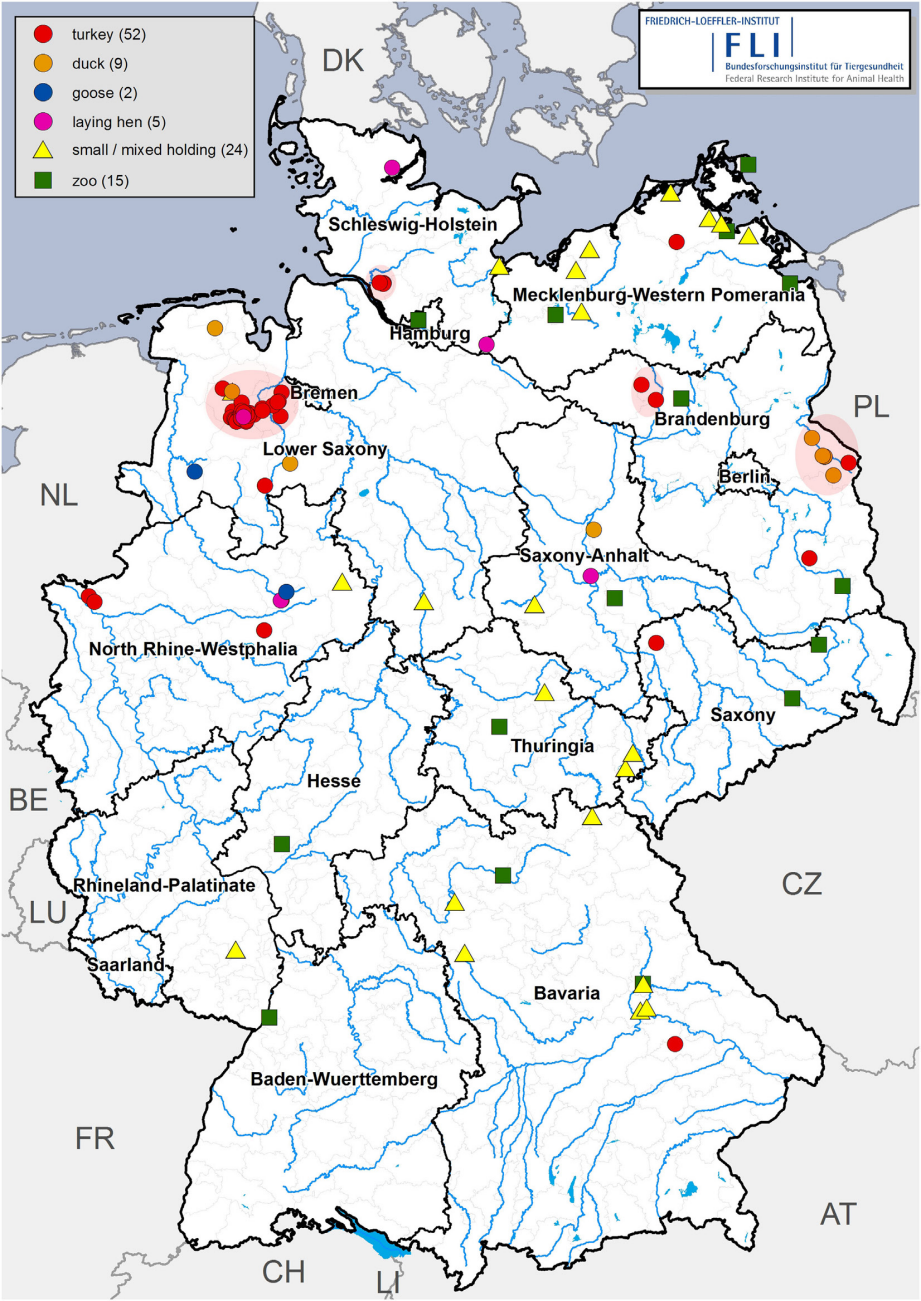
Highly pathogenic avian influenza in holdings of captive birds in Germany since November 2016. Red points: turkeys (52), orange points: ducks (9), blue points: geese (2), pink points: laying hens (5), yellow triangles: small scale, mixed holdings (24), and green squares: zoos (15). Red circles indicate outbreaks where farm-to-farm spread most likely occurred.

**Figure 4:**
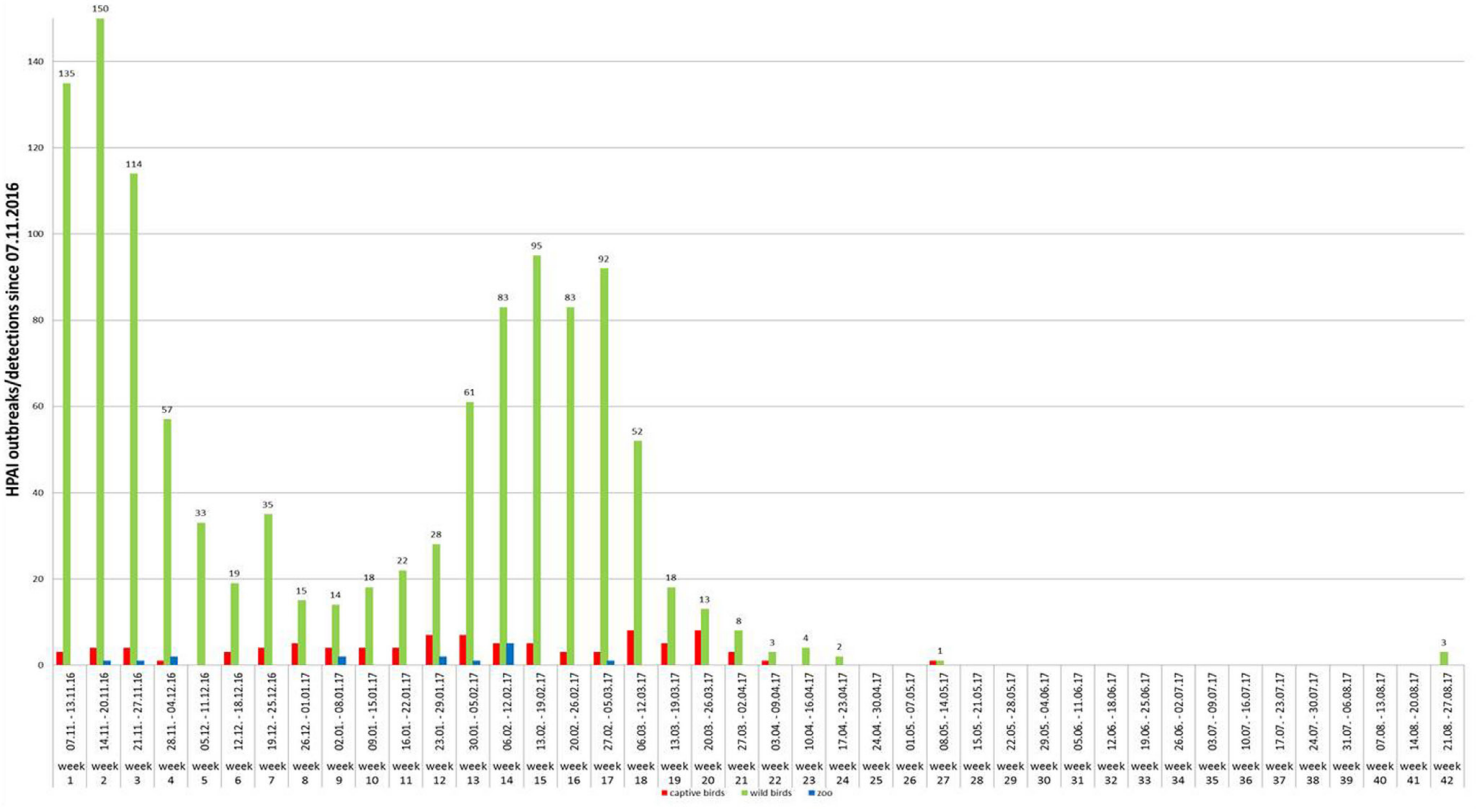
Weekly number of outbreaks of highly pathogenic avian influenza in poultry (red columns), zoos (blue columns) and cases in wild birds (green columns) in Germany (November 2016–August 2017).

Between November 8, 2016, and September 30, 2017, more than 1,150 cases of HPAI H5N8 in wild birds and 107 outbreaks in birds kept in captivity (92 poultry holdings and 15 zoos or animal parks) were reported in Germany (Figures 1, 3 and 4). The vast majority of cases in wild birds were detected in the context of passive surveillance (sick and dead birds). The last outbreak in poultry so far was reported on May 9, 2017. Thus, the HPAI epidemic seemed to be waning in Germany since April 2017 (Figure 4). Rise in ambient temperature and increasing UV radiation as well as lower densities of overwintering waterfowl on lakes and rivers may have influenced the decrease of observed cases since the tenacity of AIV is in general regarded as low (13, 14). However, in August 2017 feral mute swans in central Germany were found dead and tested positive for HPAIV H5N8.

Generally, the temporal course of the epidemic in wild birds was characterized by at least two waves, with maxima in mid-November 2016 and mid-February 2017, respectively (Figure 4). A few days after the detection of HPAIV H5N8 in wild birds, the first outbreaks were reported in non-commercial poultry (backyard) and a small animal park close to the coast of the Baltic Sea. Subsequently, large commercial poultry farms were also affected. By the end of February 2017, all federal states of Germany had reported HPAIV H5N8 infections in wild birds or poultry (Figures 1, 3 and 4). During the second wave of the epidemic, further HPAIV H5 reassortants were found in wild birds and domestic poultry (turkeys) in Schleswig-Holstein. These strains could be clearly distinguished from the first reported strains as they belonged to different genotypes involving several gene segments including another NA subtype (N5). Phylogenetic analyses indicated that multiple independent incursions of HPAIV into Germany had occurred more or less at the same time (15).

### Epidemiological Outbreak Investigations

A total of 68 commercial poultry holdings were affected by the epidemic, including 52 turkey, 5 laying hen, 9 duck, and 2 geese holdings (Figure 3). Moreover, 24 small scale, non-commercial poultry holdings were also infected by HPAIV H5N8. They were distributed almost all over Germany. Similar to the outbreaks in captive birds in zoos, they were most likely caused by primary virus incursions into the holdings/zoos *via* direct contact to infected wild birds (where captive birds were kept outdoors and with access to ponds also visited by wild birds) or *via* indirect contact (feces or material contaminated by infected carcasses). No evidence for the transmission of HPAIVs through trade of live animals, feed, or products of animal origin was detected in the course of the epidemiological outbreak investigations.

The majority of outbreaks in large commercial poultry holdings were apparently caused by single incursion events, often affecting only one out of several stables of the respective holding. In a number of cases, substantial gaps in farm biosecurity may have eased virus entry. This refers to outdoor storage of bedding material, lack of personal hygiene when entering the stables (no changing of footwear and protective clothing, lack of appropriate disinfection), regrouping of poultry flocks (mainly turkeys) during fattening, attraction of wild water birds close to the stables either by ponds or by storing silage on the premise as supply for a biogas plant. Only in the late phase of the epidemic, there was epidemiological and molecular evidence for direct farm-to-farm transmission affecting mainly turkey holdings in the area with the highest poultry densitiy, which caused approximately 25% of the total number of outbreaks (Figure 3, within red circles). The mode of farm-to-farm spread remained elusive, but was in a few cases found to be potentially related to sharing a single carcass bin by some holdings and possible vehicle contacts between farms.

Approximately 1.2 million birds died or had to be killed, and the economic losses (direct costs) were estimated as in excess of 17 million Euros.

## Discussion

Continuous cocirculation of HPAIVs and LPAIVs in poultry with frequent spill-over transmissions into migratory wild birds has been observed in several parts of Asia over more than two decades. Chances to eradicate these viruses at their source in poultry in Asia are estimated to be low. Similarly, in Egypt and West-Africa HPAIV H5N1 2.3.2.1c and HPAIV H5N8 2.3.4.4b are continuously circulating. Therefore, the poultry industry, risk managers and poultry associations must anticipate future incursions and improve their preparations for prevention and control. Fortunately, the recent HPAIV H5N8 clade 2.3.4.4a and b had no zoonotic potential, but this is prone to change as new viruses within this clade (2.3.4.4c and d) that may lead to fatal infections in mammals have already evolved in Asia (8). Efficient measures to prevent the spread of notifiable AIV include prompt detection of infection, closing affected holdings already in the case of suspected infections, immediate depopulation and cleansing/disinfection, as well as a temporary ban on restocking (7). Moreover, potential contact to wild birds, mode and frequency of farm visits, biosecurity practices, and the density of poultry holdings in a specific region are relevant risk factors for the introduction and the spread of HPAIVs (16).

Historically, HPAI outbreaks were usually geographically limited and mainly restricted to poultry, i.e., the viruses causing the outbreaks did not circulate in wild birds. This situation has fundamentally changed since the expansion of Gs/GD HPAIVs H5 to other continents, including Europe, which has led to a panzootic (5, 6). Although the epidemic of HPAIV H5N8 clade 2.3.4.4b in poultry came to a hold in late spring 2017, sporadic cases in wild water birds have continuously been reported from European countries during the summer of this year. As demonstrated by the cases detected in mute swans in central Germany in August 2017 and by several outbreaks in poultry and wild birds in Italy, Belgium, and the UK during summer 2017, continuing low level circulation among kept birds or repeated introduction into wild bird populations and vice versa cannot be excluded as long as there is the chance for direct or indirect contact to infected wild birds. This applies in particular to zoos or animal parks where birds are kept on ponds that are also frequented by wild water birds.

Adequate farm biosecurity is essential to decrease the risk of introduction and spread in poultry farms, which is particularly relevant in areas with high poultry density, particularly during epidemics. In high-risk periods and locations, losses should be compensated according to the level of biosecurity established and enforced on the affected holdings.

The most important lesson learned during the epidemic was the finding of substantial gaps in farm biosecurity and the impact of HPAI in an area of high poultry density, i.e., substantial farm-to-farm spread.

In general, protection of domestic poultry holdings from infection with HPAIV H5N8 has highest priority. Emphasis is put on the creation of a physical and functional barrier between wild bird habitats and domestic poultry holdings. Among other biosafety measures, mandatory indoor housing of poultry or the use of protected shelters (fenced and covered with fabric) minimize the risk of direct and indirect contact with infected wild birds. In particular, indirect introduction routes, e.g., through feed contaminated by wild birds, contaminated water, litter, and objects (shoes, wheelbarrows, vehicles, etc.) must be interrupted and adequate disinfection measures applied. Revision, optimization and strict implementation of biosafety measures are of utmost importance.

The HPAI H5N8 epidemic has taught the German veterinary authorities some limitations, but also the use of possible exceptions from culling as laid down in the national legislation, e.g., minimizing culling of birds kept in zoos. Based on the experience made, the national legislation is currently under revision. Furthermore, the German legislation on biosafety in poultry holdings has been amended. Not only commercial poultry farms but also small holders must now follow rules and principles that aim at reducing the risk of introduction of HPAIV into poultry farms. An online tool for an assessment of the quality of farm biosecurity by the farmers themselves is under development.

## Author Contributions

AG, CS, and FC designed the study and analyzed the data. AG and FC drafted the manuscript. CS-L, KD, TH-B, CP, JG, KD, CG, TH, ES, AP, DH, MB, and TM collected or analyzed data and edited the manuscript.

## Conflict of Interest Statement

The authors declare that the research was conducted in the absence of any commercial or financial relationships that could be construed as a potential conflict of interest.

## Footnotes

^1^https://ec.europa.eu/food/animals/animal-diseases/not-system_en

^2^http://empres-i.fao.org/eipws3g/
